# Identification of tRNA-derived small RNAs and their potential roles in porcine skeletal muscle with intrauterine growth restriction

**DOI:** 10.3389/fphys.2022.962278

**Published:** 2022-10-31

**Authors:** Mailin Gan, Jianfeng Ma, Lei Chen, Shunhua Zhang, Lili Niu, Ye Zhao, Xuewei Li, Hongmei Pan, Li Zhu, Linyuan Shen

**Affiliations:** ^1^ College of Animal Science and Technology, Sichuan Agricultural University, Chengdu, China; ^2^ Farm Animal Genetic Resource Exploration and Innovation Key Laboratory of Sichuan Province, Sichuan Agricultural University, Chengdu, China; ^3^ Key Laboratory of Pig Industry Science of Agriculture Ministry, Chongqing Academy of Animal Science, Chongqing, China

**Keywords:** IUGR, tsRNAs, skeletal muscle, IGF1, myatrophy

## Abstract

Intrauterine growth restriction (IUGR) in humans often manifests as poor growth and delayed intellectual development, whereas in domestic animals it results in increased mortality. As a novel epigenetic regulatory molecule, tRNA-derived small RNAs (tsRNAs) have been reported to be involved in many biological processes. In this study, pigs (35d) were used as a model to characterize tsRNAs by sequencing in normal and IUGR porcine skeletal muscle. A total of 586 tsRNAs were identified, of which 103 were specifically expressed in normal-size pigs and 38 were specifically expressed in IUGR pigs. The tsRNAs formed by splicing before the 5′ end anti codon of mature tRNA (tRF-5c) accounted for over 90% of tsRNAs, which were significantly enriched in IUGR pigs than in normal-size pigs. Enriched pathways of differentially expressed tsRNAs target genes mainly included metabolic pathways, Rap1 signaling pathway, endocytosis, mTOR signaling pathway, and AMPK signaling pathway. Regulatory network analysis of target genes revealed that IGF1 was one of the most important molecules of regulatory nodes in IUGR and normal porcine skeletal muscle. In addition, IGF1 was found to be one of the target genes of tRF-Glu-TTC-047, which is a highly expressed tsRNA in IUGR pigs. The findings described herein uncover the role of tsRNAs in IUGR porcine skeletal muscle development, thus providing insights into the prevention and treatment of IUGR in mammals.

## Introduction

Fetal growth and development is a complex biological process, which is affected by several factors such as genetic, environment, and maternal maturity (Lefebvre et al., 1998; Carter, 2012). Intrauterine growth restriction (IUGR) is a general term used to refer to developmental disorders of the fetus caused by maternal, fetal, or placental abnormalities, or other factors, which is mainly manifested in the newborn as decreased birth weight and organ size (Devaskar and Chu, 2016). Approximately 20% of newborns each year are affected by IUGR worldwide (de Onis et al., 1998). IUGR is an important cause of perinatal mortality and is also associated with postnatal growth and intellectual disability (Ferraz et al., 2020). In addition, IUGR may also be an early-stage disease with life-long negative consequences (Colson et al., 2021). Studies have found that IUGR newborns are more prone to developing obesity, diabetes, and cardiovascular diseases in adulthood (Kantake et al., 2020). Despite significant advancements in human medicine over the past few decades, the incidence of IUGR in neonates has not improved sufficiently.

Mammalian skeletal muscle accounts for about 40% of overall body weight and plays a significant role in motor functions (Shen et al., 2016). In addition, skeletal muscle has a powerful endocrine function, being also an important target organ for the regulation of the nervous and endocrine systems (Yin et al., 2021). Skeletal muscle development and normal function maintenance are critical to overall health. Fat and muscle tissues are often impaired when blood supply in the fetus is deficient, since blood is preferentially delivered to vital organs such as the brain and the liver (Wang et al., 2008). In addition, in the fetal blood circulation the liver is less influenced as it is the first-pass organ of umbilical venous blood, while muscle tissue is at the end point of blood circulation hence suffering greatly. Therefore, shrinking and dysplasia of skeletal muscle are the most characteristic features of IUGR in animals (Brown and Hay, 2016).

Epigenetic is a regulatory interaction between the environment and genes, which has been widely reported to be involved in the occurrence of IUGR. Epigenetic regulation in cells is related to processes such as DNA methylation (Tao et al., 2019), histone acetylation (Tian et al., 2020), and expression of non-coding RNAs (ncRNAs) (Majewska et al., 2019). As a special class of ncRNAs, tRNA-derived small RNAs (tsRNAs) were first described in 1977 and are product of tRNA degradation (Liu et al., 2021) which had long been regarded as nucleic acid garbage (Borek et al., 1977). With the development of high-throughput sequencing technology and bioinformatic analysis, tsRNAs were found to be produced under specific conditions and to play an important regulatory role (Schimmel, 2018). Compared with widely studied ncRNAs such as miRNAs, lncRNAs, and circRNAs, research on tsRNAs is still in its infancy. Thus, the mechanism of production, specific biological functions, involved regulatory networks, and expression dynamics of tsRNAs in specific tissues and under certain physiological states are still unclear.

Pigs hold an important economic value and are also an excellent animal model to study various human diseases. The incidence of IUGR in newborn piglets is approximately 15%, and circa 75% of IUGR piglets die before weaning (Che et al., 2019). In addition, IUGR piglets that survive weaning also often suffer from slow growth, poor feed utilization and meat quality throughout their productive life (Rehfeldt and Kuhn, 2006). These phenotypic characteristics are highly similar to human patients affected by IUGR, suggesting that pigs can be considered a good model for studying IUGR.

Therefore, in this study the expression characteristics of tsRNAs in IUGR-affected and normal porcine skeletal muscle were evaluated for the first time. The results discussed herein provide a reference for studying tsRNAs associated with porcine skeletal muscle development and offer new insights into understanding the role of tsRNAs in IUGR animal development.

## Materials and methods

### Ethics statement

All experimental animal procedures in this study were performed strictly in accordance with the Regulations on the Administration of Laboratory Animals (Ministry of Science and Technology of China, revised in June 2004). This experimental protocol was approved by the Animal Care and Ethics Committee of Sichuan Agricultural University (Sichuan, China, No. DKY-B20131403).

### Animals and treatment

A total of sixteen half-sib female DLY piglets (Mianyang Ming Xing Agriculture Science and Technology Development Co., Ltd. Mianyang, Sichuan, China) born from four sows were included in this study for body weight and body size analysis. Piglets born on the same day were allocated into two groups: normal (average body weight = 1.57 ± 0.07) and IUGR-affected (average body weight = 1.08 ± 0.05) pigs, after determining body weight upon birth. Three pigs (35d) whose weight after weaning was the closest to the average body weight of each group were used for obtaining samples. Left intact longissimus dorsi (LDM) and psoas major (PMM) muscle tissues were taken and weighted, and fragments of approximately 1 g in the midsection of the muscle were collected for tsRNAs sequencing.

### tsRNAs sequencing (tsRNA-seq)

Total RNA was extracted from longissimus dorsi muscle tissue for tsRNA sequencing (Shen et al., 2019). Before the sRNA library construction, the following treatments are performed to total RNA: 3′-aminoacyl (charged) deacylation to 3′-OH for 3′-adapter ligation; 3′-cP (2′,3′-cyclic phosphate) removal to 3′-OH for 3′-adapter ligation; 5′-OH (hydroxyl group) phosphorylation to 5′-P for 5′-adapter ligation; m1A and m3C demethylation for efficient reverse transcription. tsRNAs library was constructed based on methods published in previous reports (Selitsky and Sethupathy, 2015). Raw sequencing data were generated using Illumina NextSeq 500 sequencing platform.

### tsRNA-seq data analysis and target prediction

The workflow of tsRNAs-seq data processing and analysis was performed as described previously (Shen et al., 2018a). tRNA sequences of cytoplasmic origin were downloaded from Genomic tRNA Database (GtRNAdb) (Chan and Lowe, 2016). tRNA sequences of mitochondrial origin were predicted with tRNAscan-SE software (Lowe and Chan, 2016). Firstly, low-quality reads and adapter sequences were removed to obtain clean reads. Subsequently, Bowtie software was used to map clean data against pig reference genome (Sscrofa11.1) (Langmead et al., 2009). tRFs with FDR-adjusted *p* values <0.05 and fold change >2 were considered as differentially expressed. To confirm tsRNAs-seq results, the expression of the ten most expressed tsRNAs among differentially expressed tsRNA was assessed by real-time quantitative PCR (RT-qPCR). 3′ UTR sequences of pig genes were downloaded from the BioMart Ensembl tool (http://asia.ensembl.org/index.html). RNAhybrid (https://bibiserv.cebitec.uni-bielefeld.de/rnahybrid) was used for sequence alignment analysis (Krüger and Rehmsmeier, 2006). Gene Ontology (GO) and Kyoto Gene and Encyclopedia of Genomes (KEGG) pathway enrichment analyses were performed on predicted target genes.

### RT-qPCR

Quantification of tRFs and mRNAs was based on methods adopted in our previous study (Shen et al., 2018b). Total RNA was extracted from LDM tissue and porcine skeletal muscle primary cells using TRIzol reagent (Invitrogen, Guangzhou, China). tsRNAs RT-qPCR Primer Set (Tsingke Biological Technology, Chendu, China) was used to determine expression levels of selected tsRNAs with Premix Ex Taq Master Mix for RT-PCR (TaKaRa, Dalian, China) in a Bio-Rad CFX96 Real-Time PCR Detection System (Bio-Rad, Richmond, CA, United States).

### Cell culture and transfection

Porcine skeletal muscle primary cells were derived from the same batch employed in our previous study (Shen et al., 2022). Porcine primary cells were seeded in 12-well plates, and when cell confluence reached approximately 50%, tRF-Glu-TTC-047 mimic (50 nM), siIGF1 (50 nM), and the negative control (Ribobio, Guangzhou, China) were transfected into porcine primary cells using lipofectamine 3000 (Invitrogen, Guangzhou, China). Cells were harvested 24 h after transfection.

### Dual luciferase reporting system

The dual-luciferase reporter assay system was adopted based on our previous study (Shen et al., 2022). The dual-luciferase reporter system involved the amplification by PCR of the 3′ UTR of IGF1 containing an tRF-Glu-TTC-047 target site, and the insertion into the psiCHECK™-2 vector. The psiCHECK™-2 vector and the tRF-Glu-TTC-047 mimic were co-transfected into PK15 cells using lipofectamine 3000. Cells were collected after 48 h of transfection, and luciferase activity was measured using the Dual-Glo^®^ Luciferase Assay System (Promega, Madison, WI, United States) following the manufacturer’s instructions.

### Cell proliferation assays

A 0.5 cm × 0.5 cm × 1 cm tissue block was obtained from the last rib of LDM tissue and fixed with 4% paraformaldehyde. LDM tissue sections were stained with hematoxylin and eosin, and the muscle fiber cross-sectional area was calculated using ImageJ software. Cell proliferation was measured using the Cell-Light EdU Apollo567 *In Vitro* Kit (Ribobio, Guangzhou, China) following the manufacturer’s instructions, and photographed using a Nikon TE2000 microscope. Image processing was performed using the ImageJ software.

### Tissue section

The skeletal muscle tissue was fixed with 4% paraformaldehyde, dehydrated with ethanol, infiltrated and embedded with paraffin, and then sectioned (5 μm) and stained with haematoxylin and eosin (HE). Finally, use Image Pro Plus 6.0 software (National Institutes of Health, United States) to calculate the muscle fiber area (Gan et al., 2020).

### Statistical analysis

Data wrangling and statistical analysis were performed using Microsoft Excel 2016 and SPSS 22.0 (IBM, Armonk, NY, United States). Data are presented as means ± standard deviation (SD). SPSS Statistics 22.0 was used to infer statistical significance to differences between means using one-way ANOVA and the least significant difference (LSD) *post-hoc* test. Bivariate correlation analysis based on Pearson’s correlation coefficients was used to determine the relationship of body weight whith differential tsRNAs expression. *p* values ≤0.05 were considered statistically significant.

## Results

### Developmental characteristics of normal-size and IUGR pigs

**FIGURE 1:**
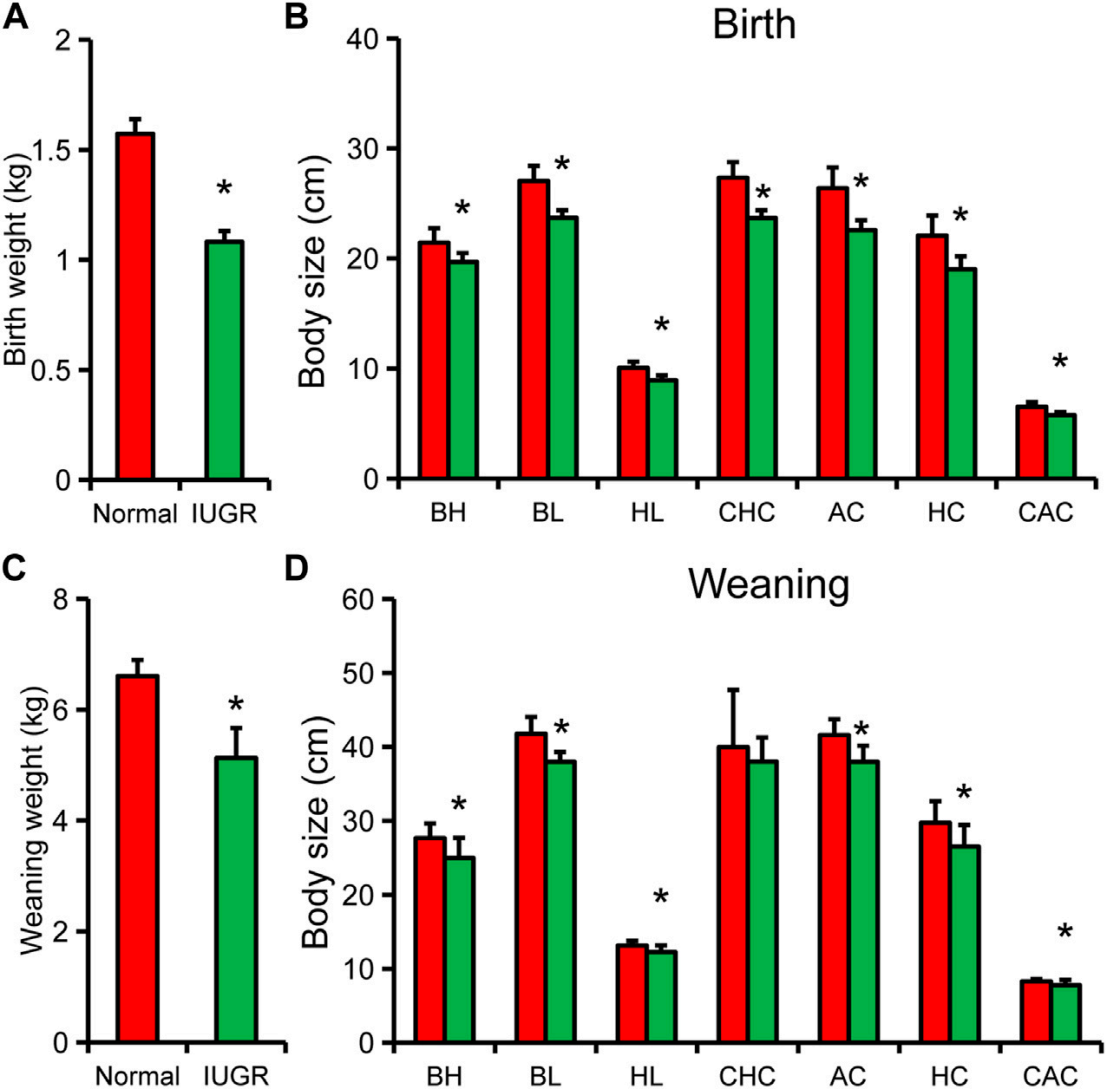
Differences in body weight and body size between intrauterine growth restriction (IUGR) and normal-size pigs. **(A)** Weight of newborn IUGR and normal-size pigs. **(B)** Body size indexes of newborn IUGR and normal-size pigs. **(C)** Body weight of IUGR and normal-size pigs at weaning. **(D)** Body size of IUGR and normal-size pigs at weaning. Results are presented as means ± SEM (N = 8); * indicates statistical significance (*p* < 0.05). BH, body height; BL, body length; HL, head length; CHC, chest circumference; AC, abdominal circumference; HC, hip circumference; CAC, cannon circumference.

Body weight and body size of newborn IUGR pigs were significantly lower than those of normal-size pigs (Figures 1A,B). Except for body height, the other body size indicators and organ indexes of IUGR pigs were reduced by 90% of those of normal pigs Figure 1B, Supplementary Figure S1. Upon weaning, body weight and other body size indicators of IUGR pigs were still significantly lower than those of normal-size pigs, except for breast circumference (Figures 1C–D). In addition, the ratio of body size index of IUGR pigs to that of normal-size pigs was further reduced, and only hip circumference was below 90% (Figure 1D).

### Characteristics of skeletal muscle of IUGR pigs

**FIGURE 2:**
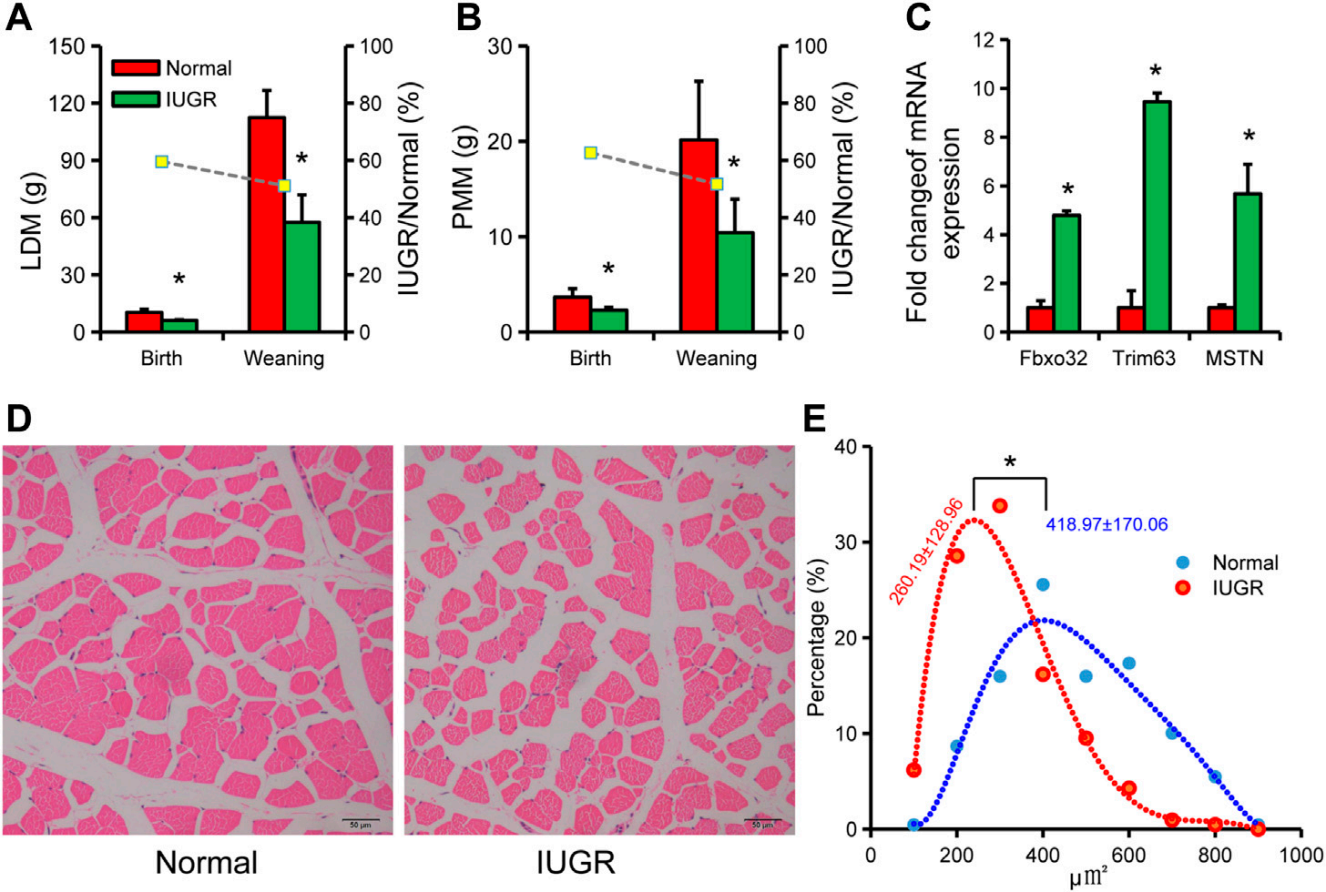
Characteristics of skeletal muscle tissue in intrauterine growth restriction (IUGR) and normal-size pigs. **(A)** Longissimus dorsi muscle (LDM) weight of IUGR and normal-size pigs at birth and weaning. **(B)** Psoas major muscle (PMM) weight of IUGR and normal-size pigs at birth and weaning. **(C)** Expression of muscle atrophy marker genes in IUGR and normal-size porcine LDM. **(D)** Cross-sectional images of LDM in IUGR and normal-size pigs. **(E)** Quantification of LDM muscle fiber area in IUGR and normal-size pigs (*n* = 3); * indicates statistical significance (*p* < 0.05).

LDM weight in IUGR pigs was significantly lower than in normal-size pigs at birth and weaning (Figure 2A). PMM weight in IUGR pigs was also significantly lower than in normal-size pigs at birth and weaning (Figure 2B). Expression of muscle atrophy marker genes *FbxO32*, *Trim63*, and *MSTN* in LDM of IUGR pigs were significantly higher than that of normal-size pigs (Figure 2C). Further analysis of skeletal muscle fibers found that the area of muscle fibers in IUGR pigs was smaller than that in normal-size pigs (Figures 2D,E).

### Expression dynamics of tsRNAs in porcine skeletal muscle

**FIGURE 3:**
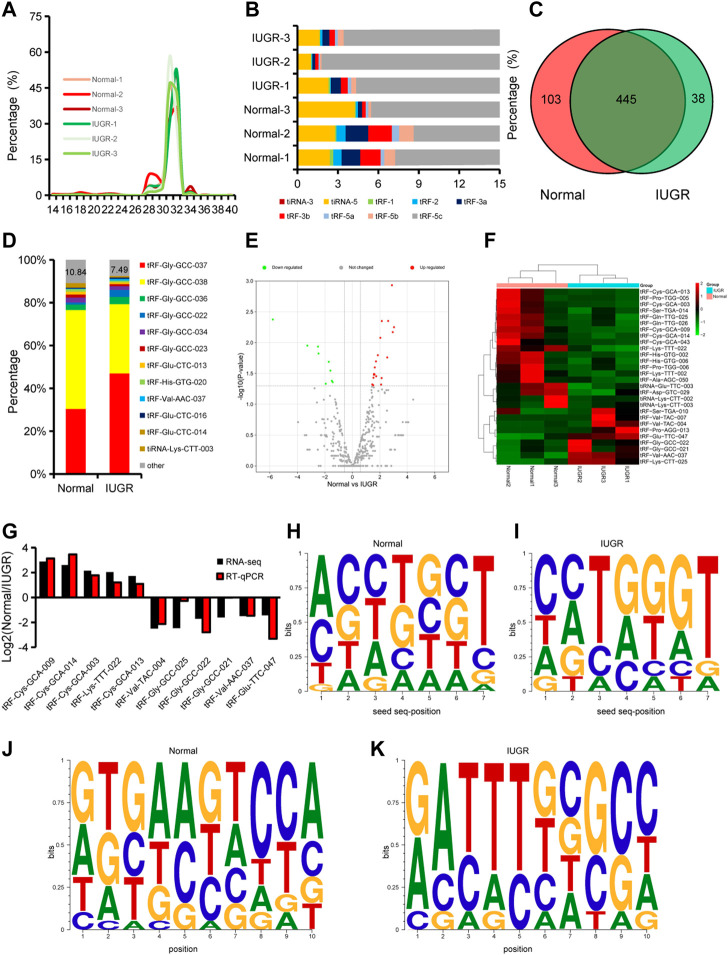
Expression dynamics of tsRNAs in porcine skeletal muscle. **(A)** Size distribution of microRNAs. **(B)** Types and distribution of tRNA-derived small RNAs (tsRNAs). **(C)** Venn diagrams with the number of expressed tsRNAs. **(D)** Composition of the top ten most expressed tsRNAs. **(E)** Volcano plot of differentially expressed tsRNAs. **(F)** Heatmap and clustering of differentially expressed tsRNAs. **(G)** Expression dynamics of selected tsRNAs by RT-qPCR. Results of at least three replicates (*n* = 3). **(H,I)** Characterization of seed sequences of differentially expressed tsRNAs highly expressed in normal-size pigs **(H)** and in intrauterine growth restriction (IUGR) pigs **(I)**. **(J,K)** Characterization of 3′-end sequences of differentially expressed tsRNAs. **(H–K)**, The size of the letter indicates the frequency of the base.

The length of tsRNAs obtained by sequencing was evaluated. The length range of tsRNAs in porcine skeletal muscle was 14–40 nt, and tsRNAs with 31 or 32 nt accounted for approximately 80% of total analyzed tsRNAs (Figure 3A). Since analyses of tRNAs is in its infancy, it would be helpful to discuss what the different classifications are of tRNAs and their purpose. In tsRNA classification, tRF-5c accounted for over 90% of identified tsRNA (Figure 3B). A total of 586 tsRNAs were identified, of which 103 were specifically expressed in normal-size pigs and 38 were specifically expressed in IUGR pigs (Figure 3C). The ten most expressed tsRNAs accounted for 89.16% of overall expression in normal-size pigs, and 92.51% in IUGR pigs (Figure 3D). Differential expression analysis showed that 28 tsRNAs were differentially expressed in LDM of IUGR and normal-size pigs, of which 19 were downregulated and nine were upregulated in IUGR (Figures 3E,F). RT-qPCR results showed that, among the top ten differentially expressed tsRNAs, only one tsRNA did not show the same expression trend identified in tsRNA-seq analysis, indicating high reliability of tsRNA-seq observations (Figure 3G). In addition, seed sequences and 3′-end sequences of differentially expressed tsRNAs were analyzed, which revealed that seed sequences of upregulated and downregulated tsRNAs differed (Figures 3H–K).

### tsRNAs target prediction, GO and KEGG pathway enrichment aanalyses

**FIGURE 4:**
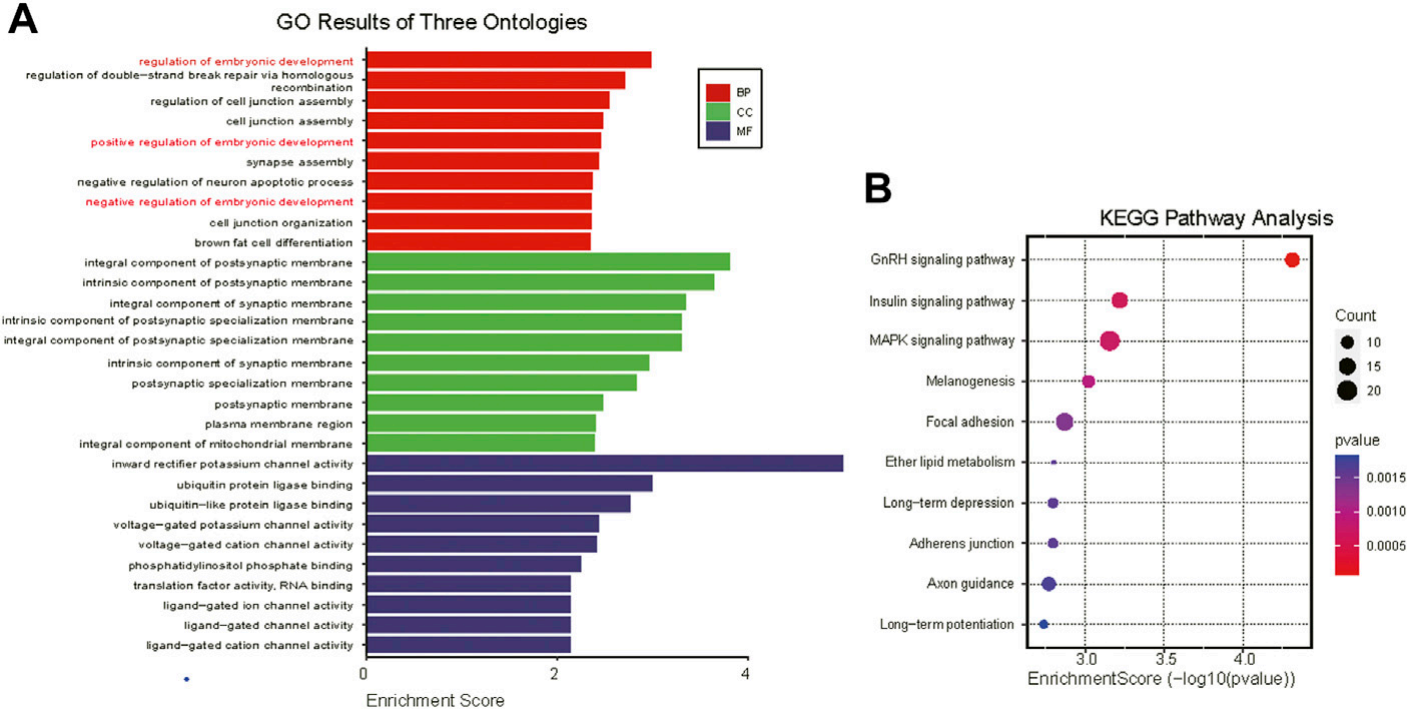
Gene Ontology (GO) and Kyoto Encyclopedia of Genes and Genomes (KEGG) pathway enrichment analyses of co-highly expressed tRNA-derived small (tsRNAs). **(A)** GO analysis of highly expressed tsRNAs target genes (only the top ten most significantly enriched signaling pathways under each item are presented) Biological Process, BP; Cellular Component, CC; Molecular Function, MF. **(B)** Bubble plot of KEGG analysis of tsRNAs target genes.

Current studies suggest that tsRNAs mainly function in a manner similar to miRNAs (Venkatesh et al., 2016). Through tsRNA-seq analysis, the four most expressed tsRNAs (which accounted for 81.42% and 84.41% of total tsRNA expression in normal-size and IUGR pigs, respectively) shared the same seed sequence (CATTGGT), thus target gene prediction was conducted for these four tsRNAs as well as for differentially expressed tsRNAs. GO and KEGG pathway enrichment analyses were conducted on the same tsRNAs. GO enrichment analysis showed that the four highly expressed tsRNAs were mainly involved in processes such as embryonic development and cell junction (Figure 4A), whereas KEGG pathway enrichment analysis showed that highly expressed tsRNAs mainly belonged to GnRH signaling pathway, insulin signaling pathway, MAPK signaling pathway, melanogenesis, and focal adhesion signaling pathways (Figures 4B).

**FIGURE 5:**
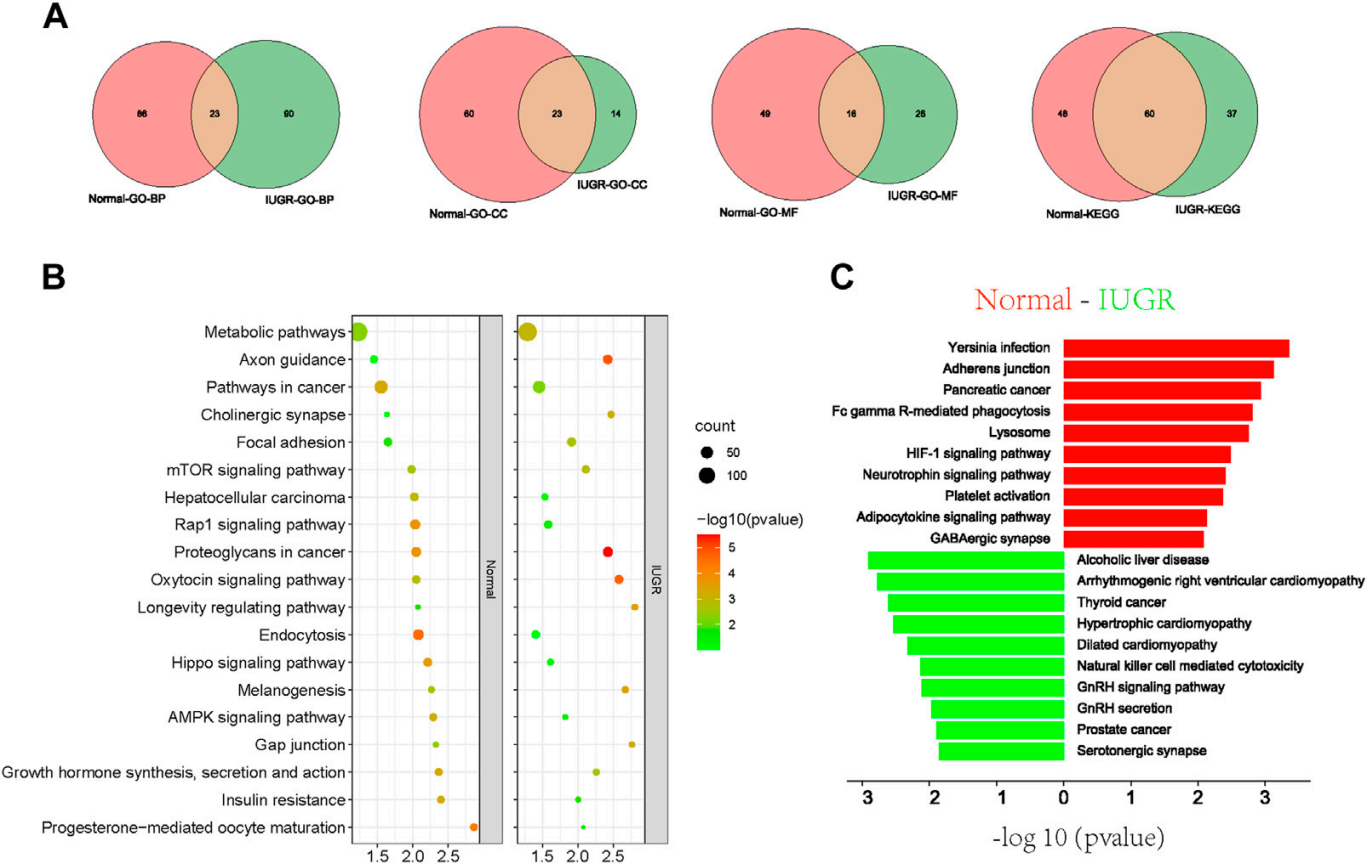
Gene Ontology (GO) and Kyoto Encyclopedia of Genes and Genomes (KEGG) pathway enrichment analyses of target genes of differential tRNA-derived small (tsRNAs). **(A)** Venn diagram of signaling pathways of differentially expressed tsRNAs in intrauterine growth restriction (IUGR) and normal-size pigs annotated against different GO and KEGG entries. **(B,C)** KEGG analysis of differentially expressed tsRNAs target genes, together with **(B)** significantly enriched signaling pathways and **(C)** individually significantly enriched signaling pathways.

In addition, analysis of target genes of differentially expressed tsRNAs revealed that upregulated or downregulated tsRNAs were commonly identified in 23, 23, and 16 pathways within the categories biological process (BP), molecular function (MF) and cellular component (CC), respectively (Figure 5A). KEGG enrichment pathway analysis showed that 60 signaling pathways were commonly identified in the two experimental groups, accounting for 61.86% and 55.56% in IUGR and normal-size pigs, respectively (Figure 5A). KEGG pathway enrichment analysis further showed that metabolic pathways, Rap1 signaling pathway, endocytosis, mTOR signaling pathway and AMPK signaling pathway were the main co-enriched signaling pathways of differential tsRNAs (Figure 5B). Among the individually enriched signaling pathways, immunity and lipid metabolism-related signaling pathways were mainly enriched in normal-size pigs, whereas cardiomyopathy and growth-related signaling pathways were mainly enriched in IUGR pigs (Figure 5C).

### IGF1 is potentially a target gene of tRF-Glu-TTC-047

**FIGURE 6:**
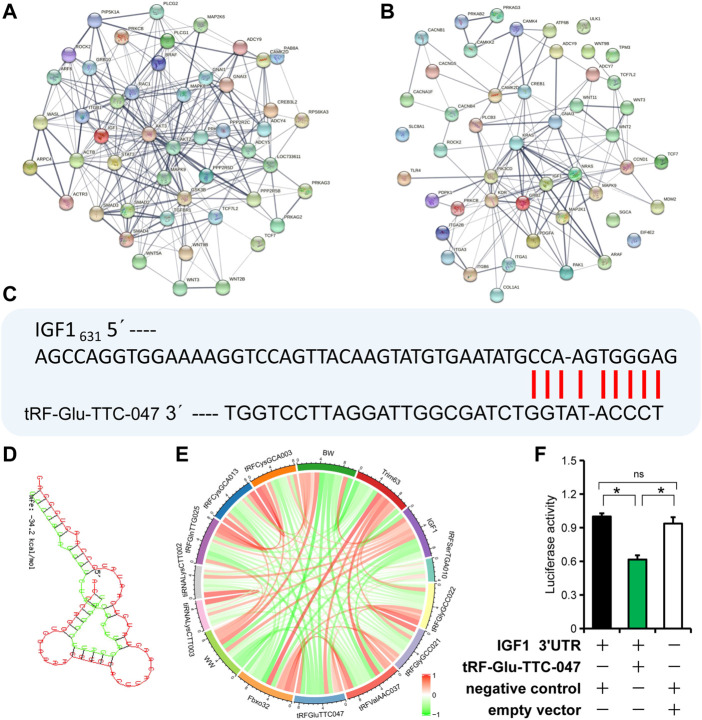
*IGF1* is one of the target genes of tRF-Glu-TTC-047. **(A)** Interaction network of upregulated tRNA-derived small (tsRNAs) target genes in normal-size pigs. **(B)** Interaction network of upregulated tsRNAs target genes in intrauterine growth restriction (IUGR) pigs. **(C)** Sequence alignment of tRF-Glu-TTC-047 with the 3′ UTR of *IGF1*. **(D)** Minimum free energy of tRF-Glu-TTC-047 and 3′ UTR of *IGF1* was predicted using RNAhybrid. **(E)** Correlation of piglet body weight (birth weight and weaning weight) with expression levels of top ten differentially expressed tsRNAs, *Fbxo32*, *Trim63*, and *IGF1*. **(F)** The dual-luciferase reporter system showed the binding relationship of tRF-Glu-TTC-047 to the 3'UTR of IGF1. n = 3; * indicates statistical significance (*p* < 0.05).

To evaluate the core molecules likely involved in the regulatory network of skeletal muscle development in IUGR pigs, genes enriched in the top twenty signaling pathways in KEGG analysis were selected and used for interaction analysis using STRING (https://cn.string-db.org/). The main core molecules in normal-size pigs were *AKT2*, *AKT3*, *ACTB*, *GSK3B*, and *ADCY4*; while the core molecules in IUGR pigs were *GRB2*, *CCND1*, *KRAS*, *KDR*, and *IGF1* (Figures 6A,B). By nucleotide sequence alignment, a binding site for tRF-Glu-TTC-047 in the 3′ UTR of *IGF1* was identified (Figures 6C,D). Moreover, correlation analysis showed a strong negative correlation between the expression of IGF1 and tRF-Glu-TTC-047 (Pearson correlation coefficient: −0.97, *p* = 0.001) (Figure 6E). Furthermore, dual-luciferase reporter system confirmed binding between tRF-Glu-TTC-047 and the 3′ UTR of IGF1 (Figure 6F).

### tRF-Glu-TTC-047 may be involved in skeletal muscle dysplasia in IUGR pigs *via* IGF1

**FIGURE 7:**
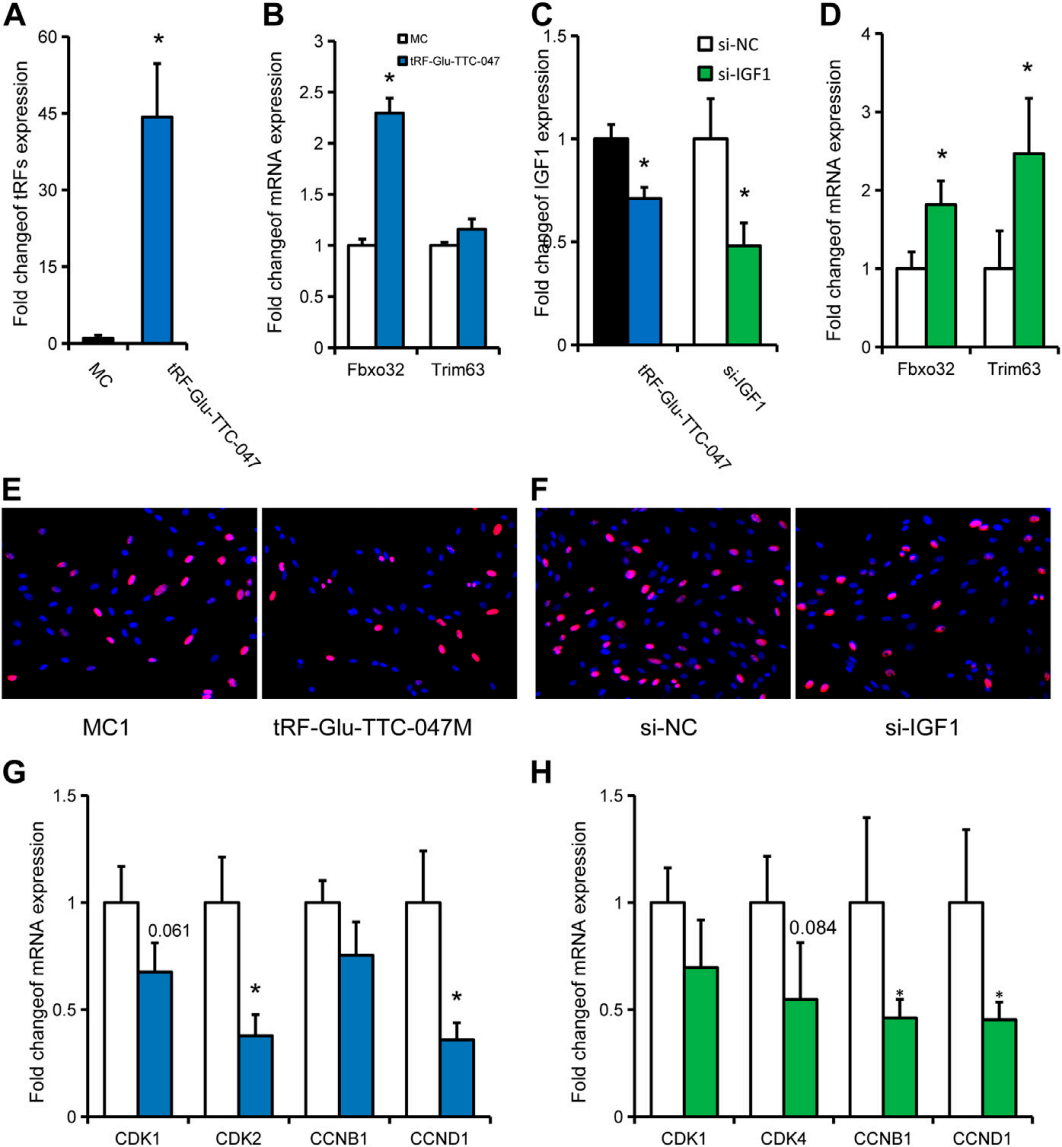
*In vitro* validation of tRF-Glu-TTC-047 observations. **(A)** Expression of tRF-Glu-TTC-047 in porcine primary cells after transfection with tRF-Glu-TTC-047 mimic. **(B)** Expression levels of Fbxo32 and Trim63 in porcine primary cells after transfection with tRF-Glu-TTC-047 mimic. **(C)** Expression levels of IGF1 in porcine primary cells after transfection with tRF-Glu-TTC-047 mimic and si-IGF1. **(D)** Expression levels of Fbxo32 and Trim63 in porcine primary cells after transfection with si-IGF1. **(E,F)** Edu proliferation assay after transfection of (E) tRF-Glu-TTC-047 mimic and **(F)** si-IGF1 **(G)** Expression of proliferation-related genes in porcine primary cells transfected with tRF-Glu-TTC-047 mimic. **(H)** Expression of proliferation-related genes in porcine primary cells transfected with si-IGF1. Results are presented as mean ± SEM of at least three replicates (*n* = 3); * indicates statistical significance (*p* < 0.05).

Then, tRF-Glu-TTC-047 was used for *in vitro* cell validation of the above-discussed observations. Overexpression of tRF-Glu-TTC-047 was obtained in primary porcine skeletal muscle cells by transfecting a synthetic tRF-Glu-TTC-047 mimic (Figure 7A), which significantly promoted the expression of *Fbxo32* in porcine primary cells (Figure 7B), while significantly inhibited the expression of its target gene *IGF1* (Figures 7B,C). Transfection of si-IGF1 successfully interfered with the expression of *IGF1* in primary porcine cells, resulting in significant upregulation of *Fbxo32* and *Trim63* (Figures 7C,D). Considering that a large number of cell proliferation-related pathways were independently enriched in IUGR pigs, and CCND1 as a core molecule was involved in the regulatory network of skeletal muscle development in IUGR pigs, the effect of transfection of tRF-Glu-TTC-047 mimic on the proliferation of porcine primary cells was evaluated. Transfection of tRF-Glu-TTC-047 mimic and si-IGF1 inhibited the positive rate of Edu cells (Figures 7E,F). Correspondingly, transfection of tRF-Glu-TTC-047 mimic significantly inhibited the expression of *CDK2* and *CCND1* in cells (Figure 7G), whereas transfection of si-IGF1 also significantly inhibited *CCNB1* and *CCND1* expression (Figure 7H).

## Discussion

In recent years, tsRNAs have been considered a new class of ncRNAs with important biological functions (Li et al., 2021). Growing evidence suggests that tsRNAs play important roles in a variety of biological processes and can be considered as potential disease biomarkers and therapeutic targets (Chen et al., 2021). Studies on tsRNAs are still incipient and mainly focus on cancer and cardiovascular diseases, with still few studies in other research fields (Zong et al., 2021). IUGR is an important condition that affects humans and livestock, negatively impacting animal production. In humans, approximately 30 million of infants are affected by IUGR each year worldwide (Shen et al., 2018a). Similarly, 10%–25% of newborn piglets in pig production are classified as IUGR piglets (Klaaborg and Amdi, 2020). In IUGR animals, skeletal muscle is one of the organs most affected by altered blood circulation and perfusion. Herein, tsRNAs in normal-size and IUGR porcine skeletal muscle were characterized by high-throughput sequencing (Năstase et al., 2018). In addition, functional analysis of canonical tsRNAs was performed *in vitro*. These findings contribute to further the understanding of the role of tsRNAs in skeletal muscle development and provide new insights to foster IUGR-related research.

### Characterization of tsRNA in skeletal muscle of normal-size and IUGR pigs

Highly abundant tsRNAs are more likely to play an important role in maintaining normal physiological functions. The top ten highly expressed tsRNAs in normal-size pigs were tRF-Gly-GCC-038, tRF-Gly-GCC-037, tRF-Gly-GCC-036, tRF-Gly-GCC-034, tRF-Gly-GCC-023, tRF-Glu-CTC-013, tiRNA-Lys-CTT-003, tRF-His-GTG-020, tRF-Gly-GCC-022, and tRF-Glu-CTC-014, of which the four most expressed tsRNAs (accounting for 81.42% of total tsRNA abundance) were shown to contain an identical seed sequence. In IUGR pigs, eight of the ten most expressed tsRNAs were also identified in normal-size pigs, and four tsRNAs (accounting for 84.41% of total tsRNAs) with the same seed sequence. This expression pattern is the same as that of normal pigs. Thus, these results indicate tsRNA expression specificity. Since tsRNAs are derived exclusively from tRNAs after cleavage at specific sites, sequence similarity is higher than that observed in miRNAs; therefore, several tsRNAs contain similar seed sequences (Venkatesh et al., 2016; Rashad et al., 2020).

TsRNAs can be divided into two categories, namely tRNA-halves (tiRNAs) and tRNA-derived small fragments (tRFs) (Venkatesh et al., 2016). tiRNAs are generated by cleavage of anticodon loops and are further divided into ti-RNA3 and ti-RNA5 according to 5′ and 3′. tRFs can be divided into tRF1, tRF2, tRF3, and tRF5 according to their breaking sites. tRFs can be divided into tRF1, tRF2, tRF3, and tRF5 according to their breaking sites, among which tRF3 can be further divided into tRF3a and tRF3b, and tRF5 can be further divided into tRF5a, tRF5b, and tRF5c (Martinez, 2018). In this study, it was found that tsRNAs in porcine skeletal muscle were mainly tRF5c type, accounting for more than 90%. These distribution characteristics of tsRNA in skeletal muscle are similar to other previous reports (Pawar et al., 2020), suggesting that a small number of tsRNAs play an important role in maintaining normal physiological functions.

### Function prediction of tsRNAs

Studies have shown that tsRNAs exist widely in bacteria, fungi, animals, and plants. tsRNAs exert biological functions through a variety of mechanisms, including by regulating mRNA stability or translation, or acting as epigenetic regulators (Kumar et al., 2016). tsRNAs’ most studied biological functions are those similar to those exerted by miRNAs. tsRNAs bind to target genes through a seed sequence (positions 2−8 at the 5′ end of the molecule) to inhibit the expression of the target gene. CU1276 is one of the first tsRNAs reported to bind target genes through seed sequences; CU1276 was found to bind to the 3′ UTR of *RPA1*, thus inhibiting its expression and consequently the proliferation of B-cell lymphoma cells (Maute et al., 2013). Subsequent studies have reported that tRF/miR-1280 inhibits stem-like cells and metastasis in colorectal cancer by targeting *JAG2* (Huang et al., 2017). In addition, the 5′ tiRNA-His-GTG was shown to promote colorectal cancer progression by targeting *LATS2* expression (Tao et al., 2021). Considering these studies, target gene prediction and functional enrichment analysis were conducted based on seed sequences of tsRNAs (Shen et al., 2018b). Interestingly, 586 different tsRNAs shared 221 seed sequences, and the four most expressed tsRNAs accounted for over 80% of total tsRNA abundance but uniquely shared a single seed sequence. The highly expressed tsRNAs were mainly involved in the biological process of embryonic development and cell connection, and most significantly enriched pathways were GnRH signaling pathway, insulin signaling pathway, MAPK signaling pathway, melanogenesis, and focal adhesion signaling pathways. The GnRH signaling pathway (Korpysz and Szalecki, 2019), insulin signaling pathway (Dunlop et al., 2021), and MAPK signaling pathway (Choi et al., 2020) have been reported to be associated with IUGR. In addition, differentially expressed tsRNAs in normal-size and IUGR porcine skeletal muscle were mainly enriched in metabolic pathways, Rap1 signaling pathway, endocytosis, mTOR signaling pathway, and AMPK signaling pathway. It is known that energy metabolism and endocrine abnormalities are the main factors leading to IUGR (Sharma et al., 2017), and the mTOR signaling pathway has been considered the core signaling pathway involved in IUGR (Xu et al., 2020). In addition, signaling pathways and biological processes enriched with differentially expressed tsRNAs and highly expressed tsRNAs highly overlapped. These biological processes and signaling pathways are likely to play an important role in maintaining normal skeletal muscle function, and differentially expressed tsRNAs annotated to separate signaling pathways can be considered potential IUGR biomarkers.

In addition to exerting biological functions in a similar fashion to miRNAs, tsRNAs have also been reported to act in a motif-like manner through specific sequences at 3′ end (Wang et al., 2006). Thus, sequence signatures of seed sequences or 3′ ends of tsRNAs upregulated or downregulated in IUGR porcine skeletal muscle were analyzed. Xie et al. carried out comparative analysis of human, mouse, rat and dog genomes, and created a systematic catalog of common regulatory motifs in promoters and 3′ untranslated regions (3′ utr) to identify all functional elements encoded in the human genome (Xie et al., 2005). By sequence alignment analysis, the germplasm sequences of upregulated and downregulated tsRNAs in IUGR pigs matched cluster28 and cluster52, respectively. The 3′ end sequences of upregulated tsRNAs could potentially match cluster10, while no matching motifs were found for downregulated tsRNAs (Xie et al., 2005). These results suggest that tsRNAs also have similar characteristics to miRNAs in the regulation of gene expression.

tsRNAs may also form intermolecular RNA G tetramers (RG4) through a sequence of four to five guanine residues at the 5′ end which affect translation initiation through the translation initiation factor eIF4F complex (Lyons et al., 2017). However, since current research on tsRNAs is still incipient, therefore their mechanism of action and regulatory network are still unknown, and further studies are needed.

### Construction and improvement of tsRNAs regulatory network

In this study, the most well-established patterns in functional studies of tsRNAs (similar to miRNAs) were adopted, and a tsRNA (i.e., tRF-Glu-TTC-047) that was found upregulated in IUGR porcine skeletal muscle was selected preliminary functional validation of the findings (Jia et al., 2020). tRF-Glu-TTC-047 had a higher expression level and the highest negative correlation with piglet body weight. Enrichment pathway analysis of tRF-Glu-TTC-047 target genes showed that those were mainly related to cell proliferation (Burton and Jauniaux, 2018) and immunity (Tao and Dahl, 2013), which were highly similar to the physiological disturbances observed in IUGR-affected animals. Further screening revealed that *IGF1* is likely among tRF-Glu-TTC-047 target genes, with *IGF1* at the core of IUGR porcine skeletal muscle regulatory network. *IGF1* has also been reported to be involved in the regulation of skeletal muscle development and associated with the occurrence of IUGR (Zhu et al., 2022). The binding relationship between the 3′ UTR of *IGF1* and tRF-Glu-TTC-047 was further confirmed by a dual-luciferase reporter system. Moreover, *in vitro* experiments showed that overexpression of tRF-Glu-TTC-047 and interference with *IGF1* could have similar effects on porcine primary skeletal muscle cells, i.e., promoting muscle atrophy (Schiaffino et al., 2013) and inhibiting cell proliferation (Hakuno and Takahashi, 2018).

**FIGURE 8:**
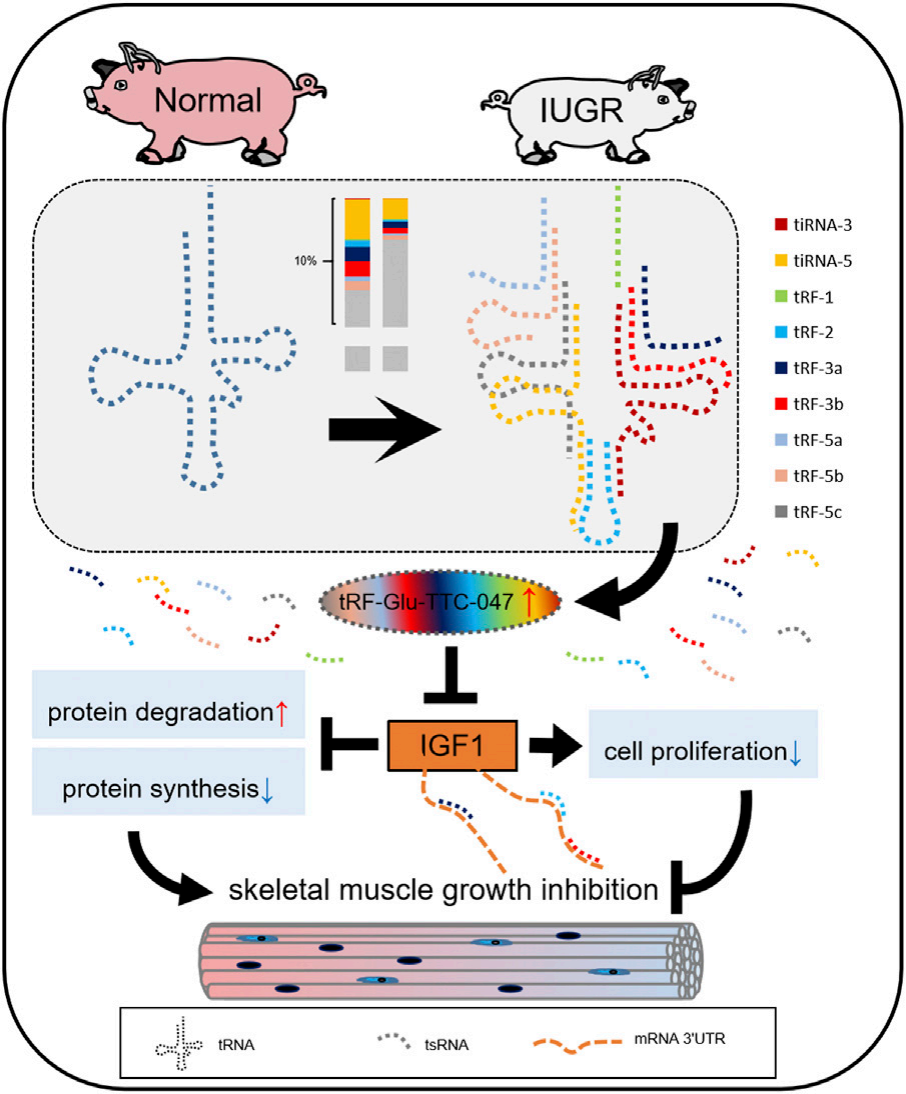
Simplified diagram of the regulatory network of tRF-Glu-TTC-047 involved in skeletal muscle development in intrauterine growth restriction (IUGR) pigs by targeting IGF1. Different color bars correspond to different types of tsRNA.

Based on the above-described findings, a schematic diagram has been proposed for the role of tRF-Glu-TTC-047 in the regulation of skeletal muscle development. Future studies will involve refining the regulatory network centered on tRF-Glu-TTC-047 and exploring its role in other physiological and disease states (Figure 8).

## Conclusion

The present study is the first to report the expression profile of tsRNAs in normal-size and IUGR porcine skeletal muscle tissue. A total of 586 tsRNAs were identified which mainly belonged to the tRF5c type. Distribution of tsRNAs expression was remarkably uneven, and two of the most expressed tsRNAs accounted for over 75% of total tsRNA abundance. In addition, tRF-Glu-TTC-047 was shown to be a highly expressed tsRNA in IUGR porcine skeletal muscle which might be involved in skeletal muscle development by targeting *IGF1*. Our results provide a reference for the study of tsRNAs in skeletal muscle and offer new insights into understanding the impacts of IUGR on skeletal muscle in animals.

## Data Availability

The datasets presented in this study can be found in online repositories. The names of the repository/repositories and accession number(s) can be found below: https://www.ncbi.nlm.nih.gov/, PRJNA800654.

## Supplementary material

Supplementary Figure 1 Tissue weight characteristics of normal and IUGR pigs.
