# Supplementary figures and images for: Identification of tRNA-derived small RNAs and their potential roles in porcine skeletal muscle with intrauterine growth restriction
Source: Front Physiol. 2022 Oct 31;13:962278. doi: 10.3389/fphys.2022.962278 (PMC9662792; doi:10.3389/fphys.2022.962278)

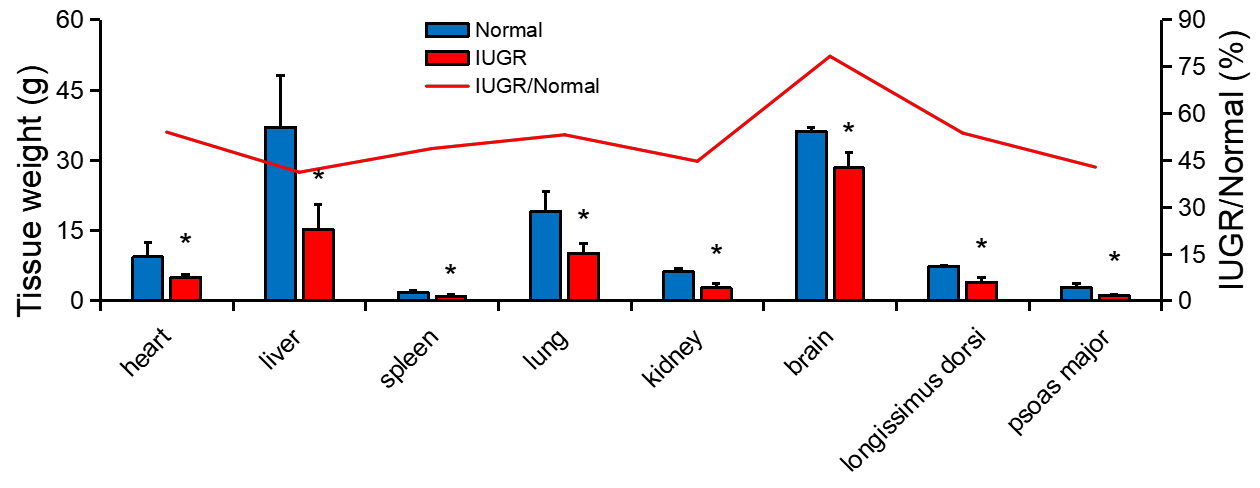

Supplement: Supplementary file 1 [file Image1.tif]
